# Supplementary figures and images for: The effects of rifaximin and lactulose on the gut-liver-brain axis in rats with minimal hepatic encephalopathy
Source: PLoS One. 2025 Jun 17;20(6):e0325988. doi: 10.1371/journal.pone.0325988 (PMC12173377; doi:10.1371/journal.pone.0325988)

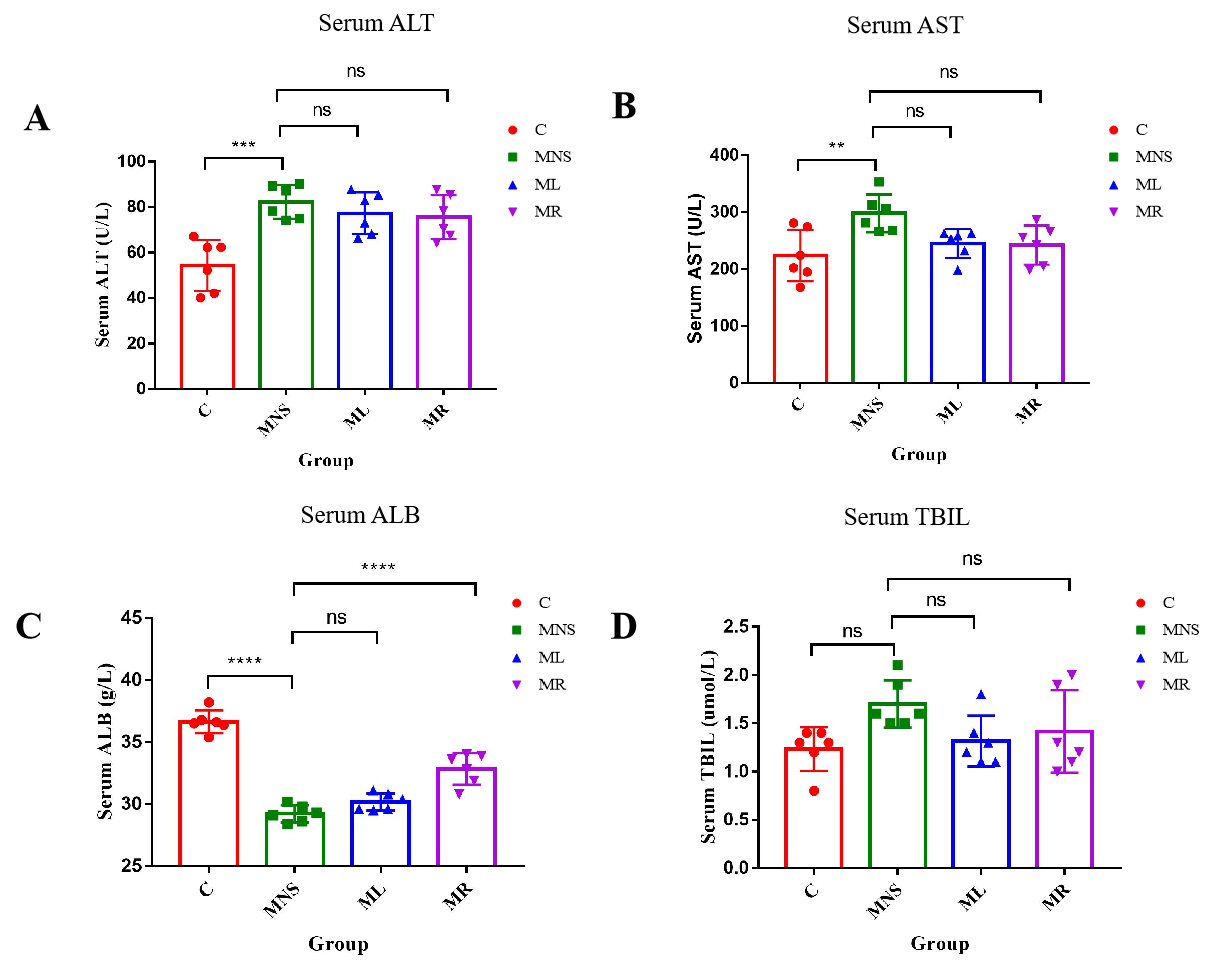

Supplement: S1 Fig — (A) Serum ALT, (B) AST, (C) ALB, and (D) TBIL levels were assessed as indicators of liver injury. (PNG) [file pone.0325988.s001.png]

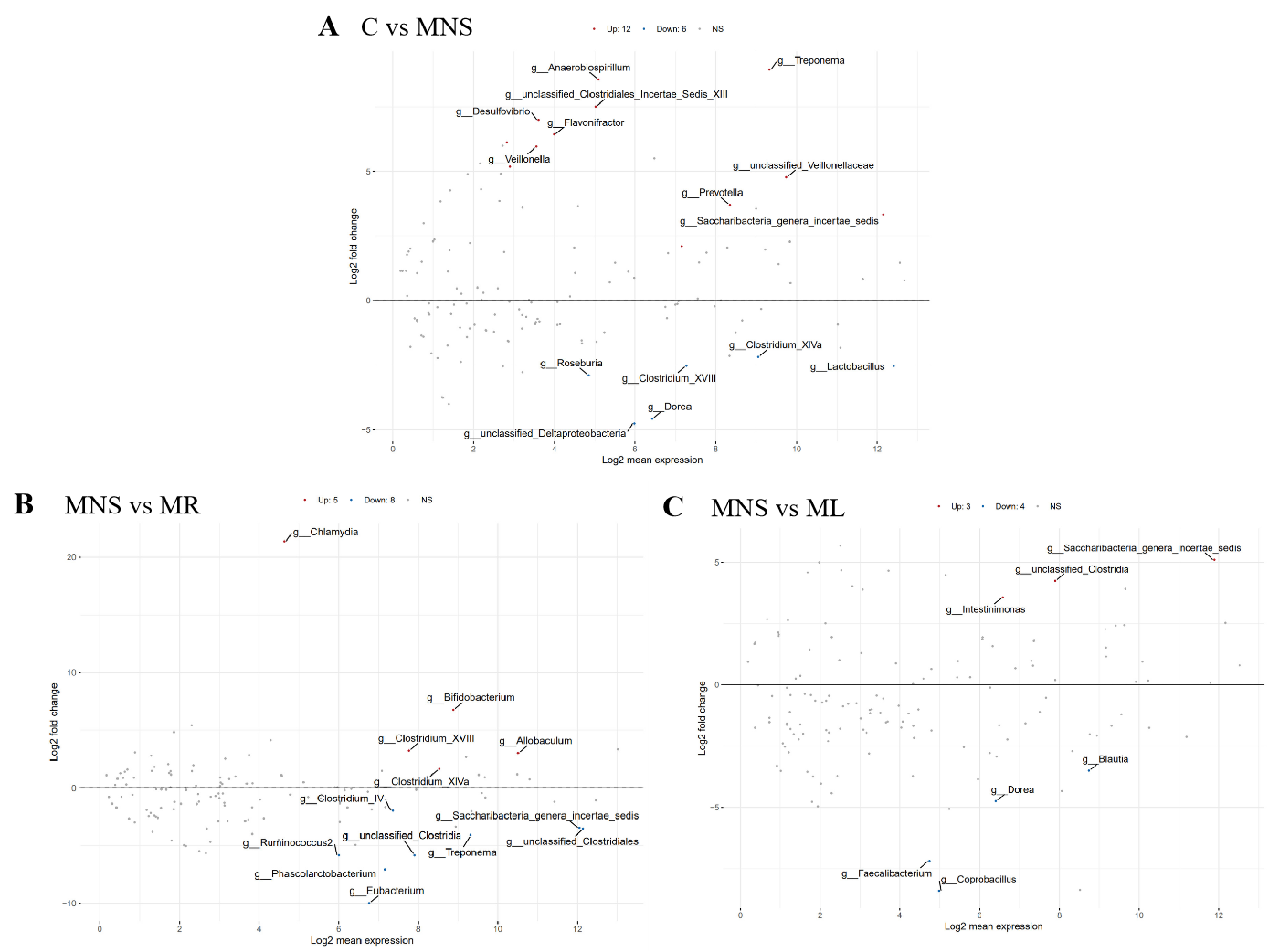

Supplement: S2 Fig — (A) Changes in relative abundance of the gut microbiota between C and MNS group. (B) Changes in relative abundance of the gut microbiota between MNS and MR group. (C) Changes in relative abundance of the gut microbiota between MNS and ML group. (PNG) [file pone.0325988.s002.png]

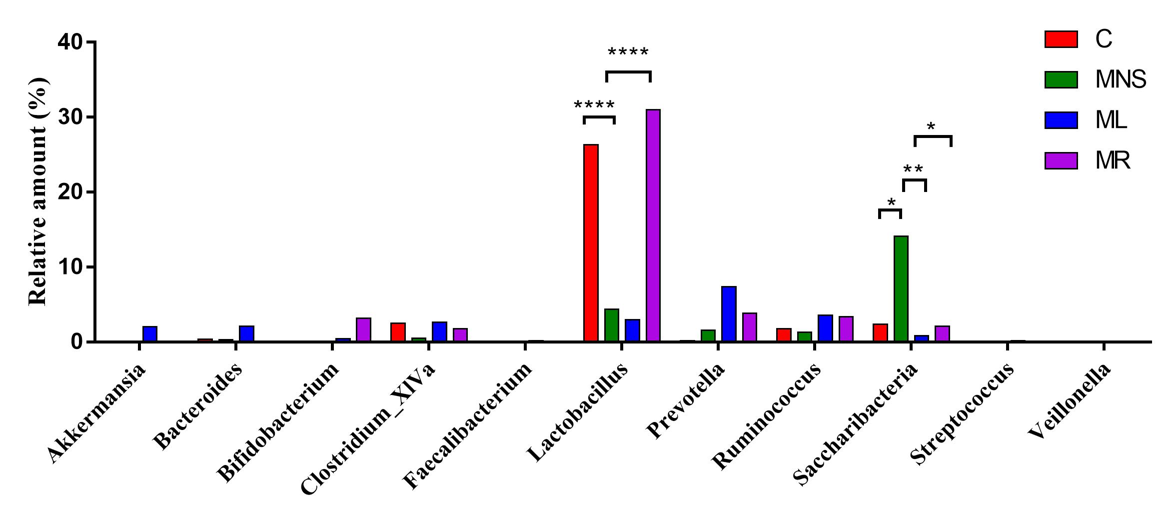

Supplement: S3 Fig — (PNG) [file pone.0325988.s003.png]

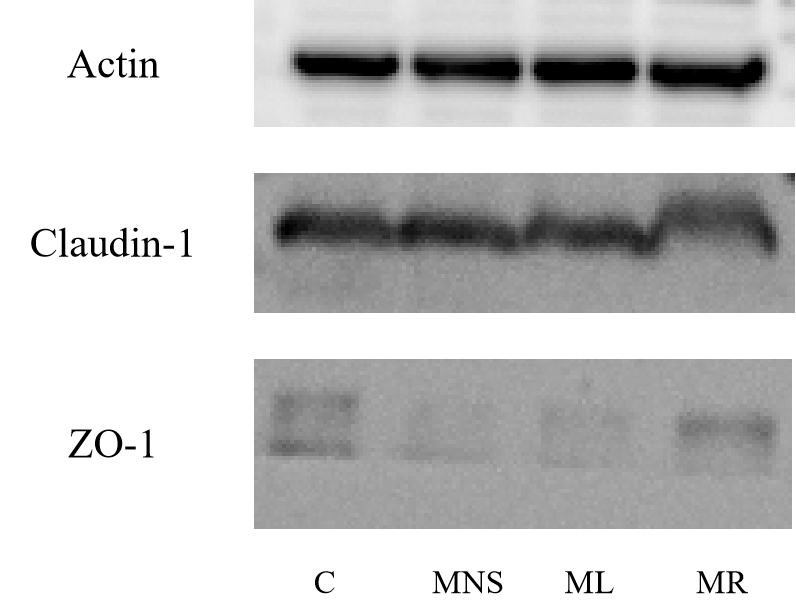

Supplement: S4 Fig — (PNG) [file pone.0325988.s004.png]

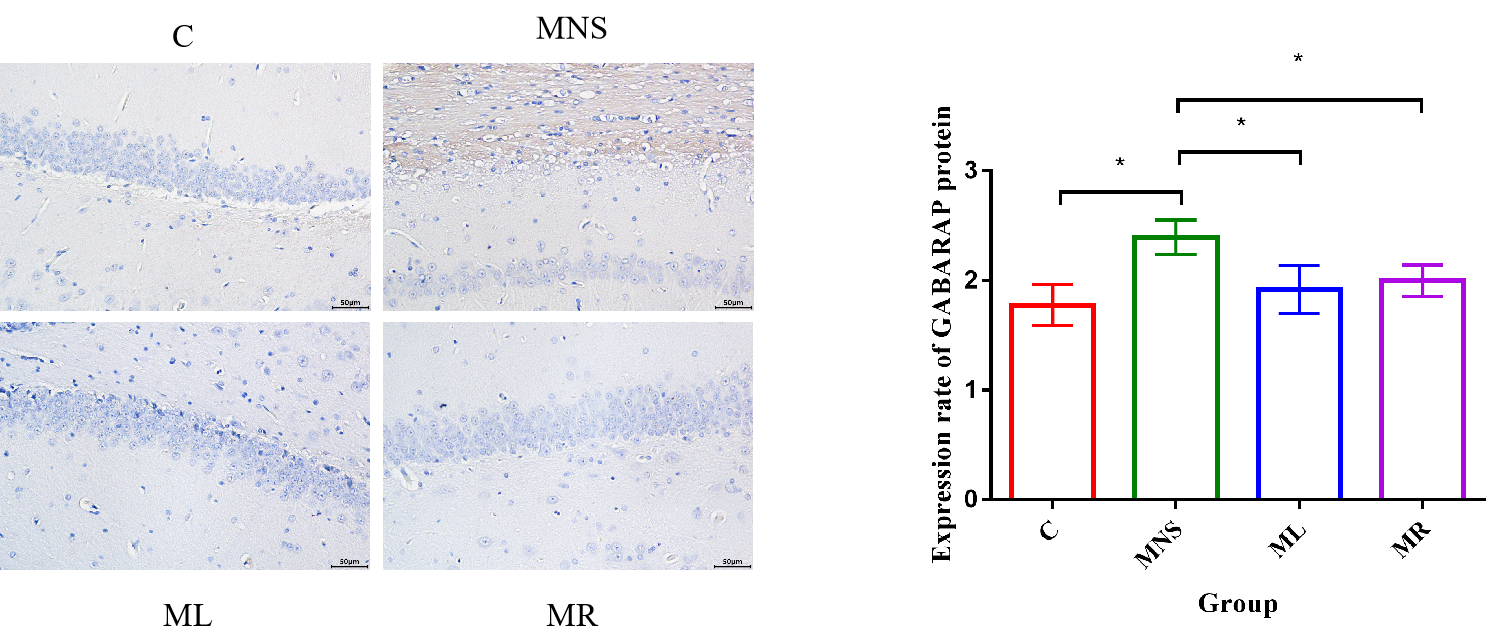

Supplement: S5 Fig — (PNG) [file pone.0325988.s005.png]
