# Supplementary material for: The effects of rifaximin and lactulose on the gut-liver-brain axis in rats with minimal hepatic encephalopathy
Source: PLoS One. 2025 Jun 17;20(6):e0325988. doi: 10.1371/journal.pone.0325988 (PMC12173377; doi:10.1371/journal.pone.0325988)
Supplement: S1 Table — (DOCX) [file pone.0325988.s006.docx]

**Supplementary table 1. MHE diagnosis and escape latency after model building.**

| Group | Latency of BAEP I (ms) | Incidence of MHE (%) | Escape latency (s)  (After building, test 4) |
| --- | --- | --- | --- |
| Control model | 1.28±0.04 | 0.00 (0/6) | 7.67±4.60 |
| MHE model | 1.54±0.05* | 90.00 (18/20) | 30.09±12.00* |

Abbreviations: BAEP, brainstem auditory evoked potentials; MHE, mild hepatic encephalopathy. Results are shown as the means ± SD. * *p*<0.05 vs control model using a Mann-Whitney U test.
