# Supplementary material for: The effects of rifaximin and lactulose on the gut-liver-brain axis in rats with minimal hepatic encephalopathy
Source: PLoS One. 2025 Jun 17;20(6):e0325988. doi: 10.1371/journal.pone.0325988 (PMC12173377; doi:10.1371/journal.pone.0325988)
Supplement: S1 File — (PDF) [file pone.0325988.s007.pdf]

occludin

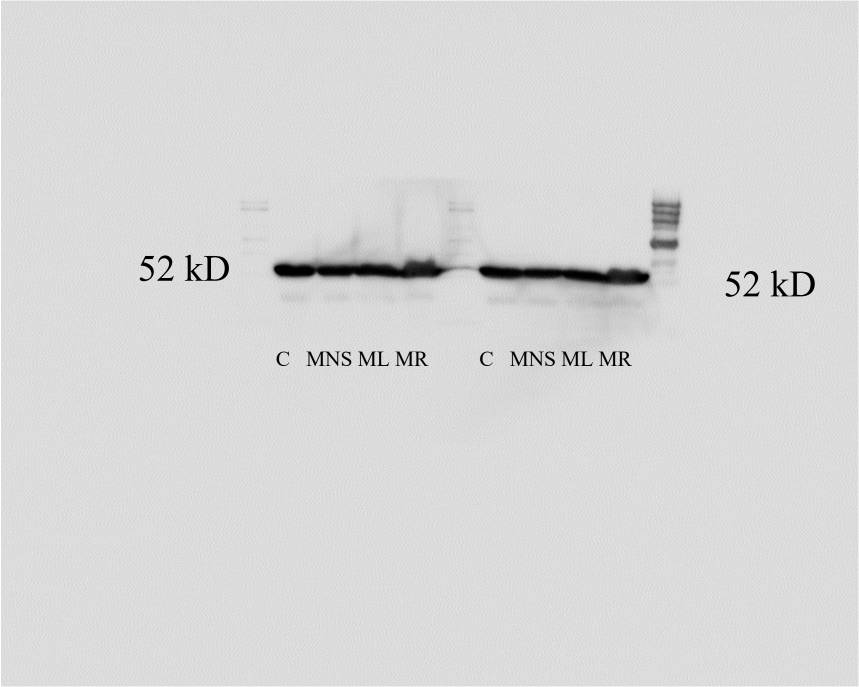

small intestine

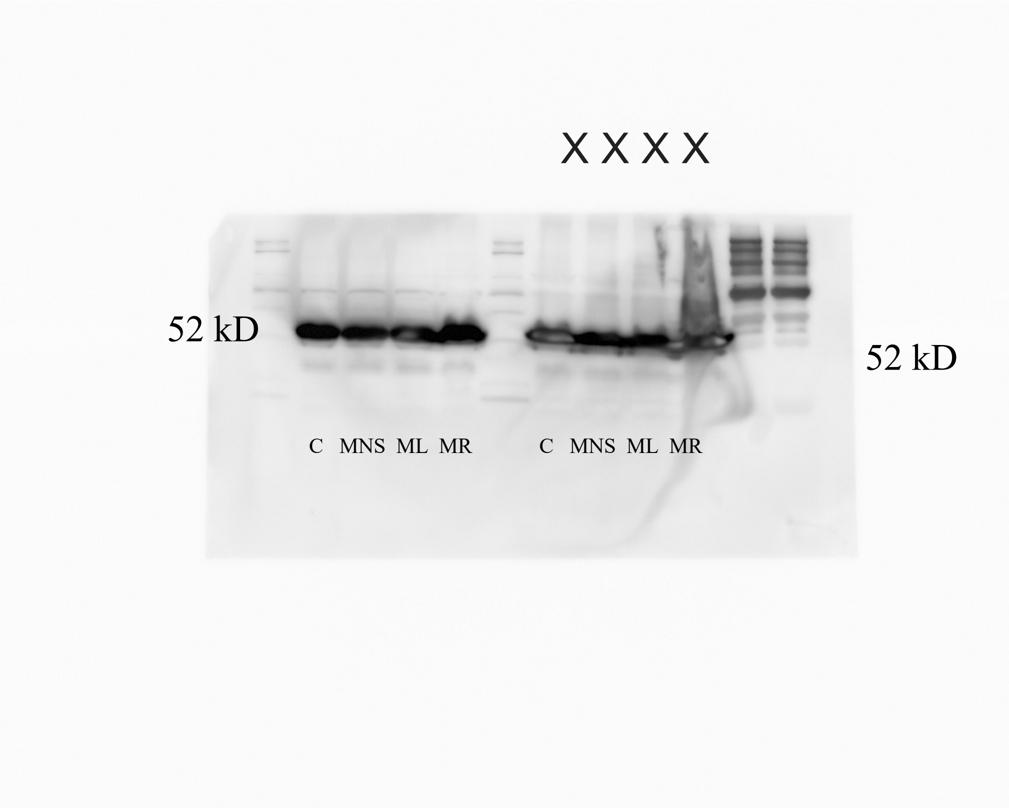

small intestine

Actin

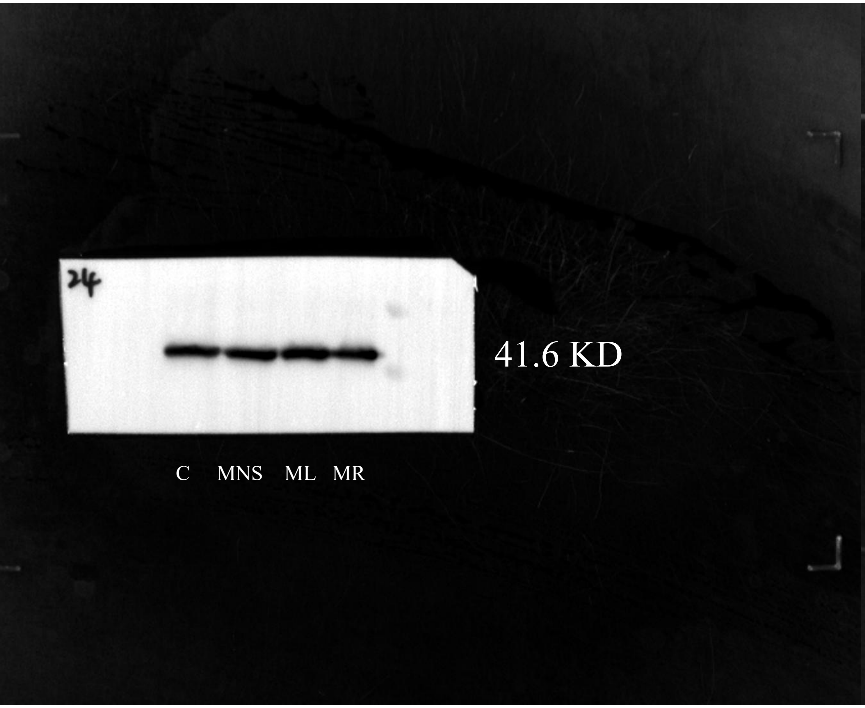

small intestine

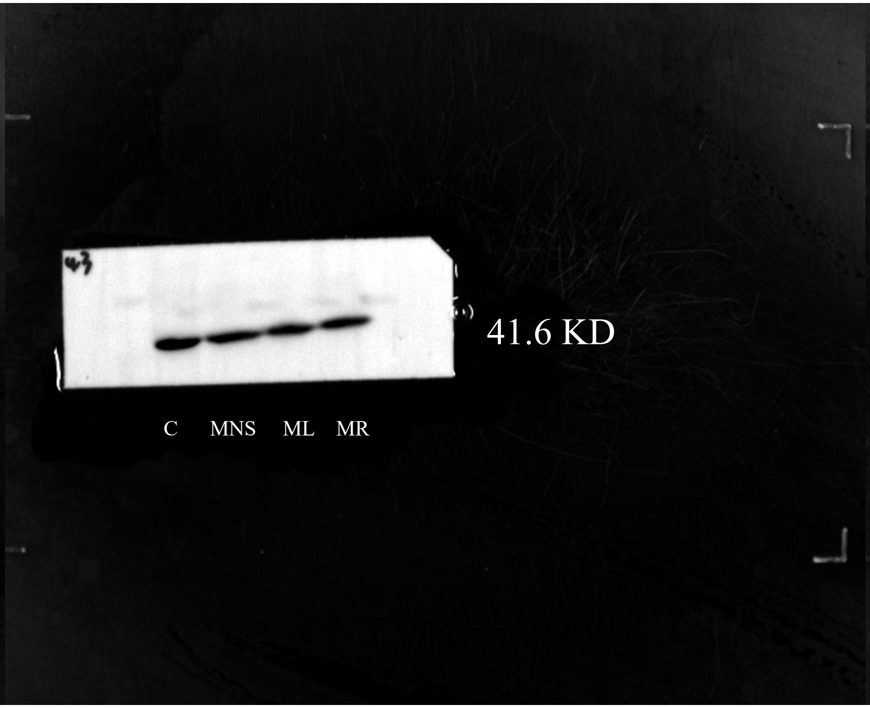

small intestine

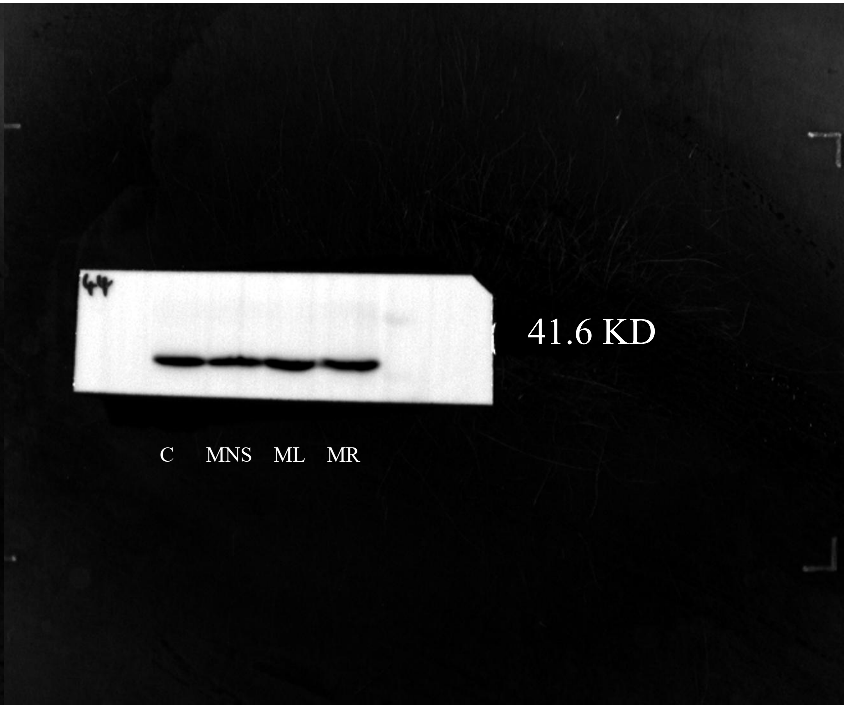

small intestine

Claudin-1

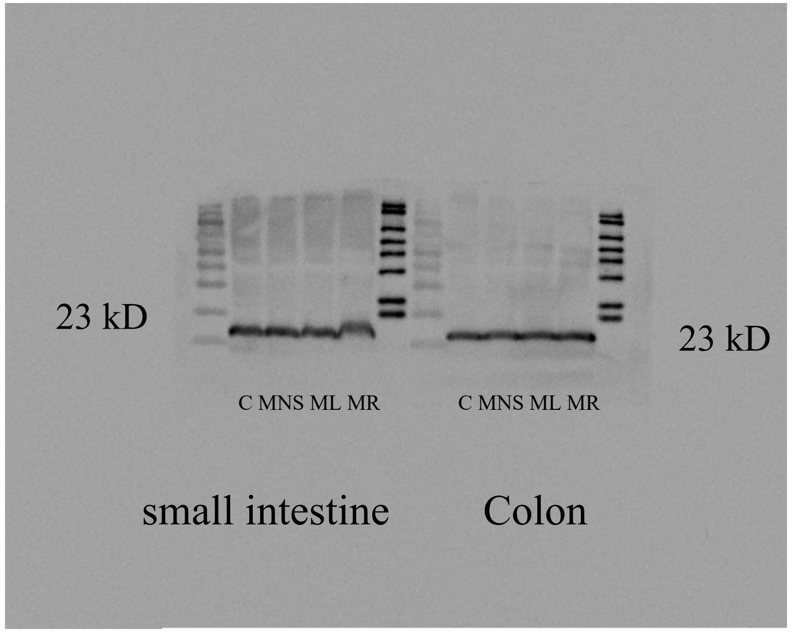
23 kD
23 kD
C MNS ML MR
C MNS ML MR
small intestine
Colon

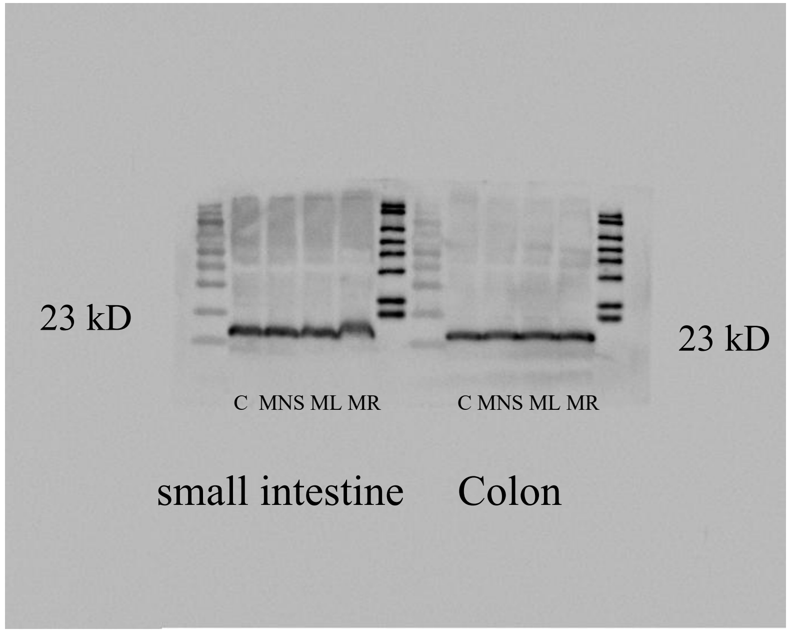
23 kD
23 kD
C MNS ML MR
C MNS ML MR
small intestine
Colon

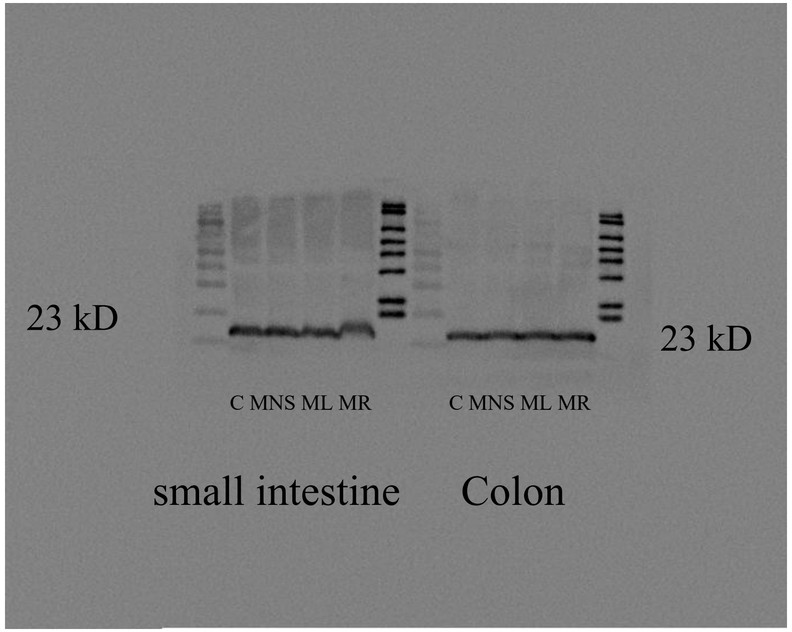
23 kD
23 kD
C MNS ML MR
C MNS ML MR
small intestine
Colon

ZO-1

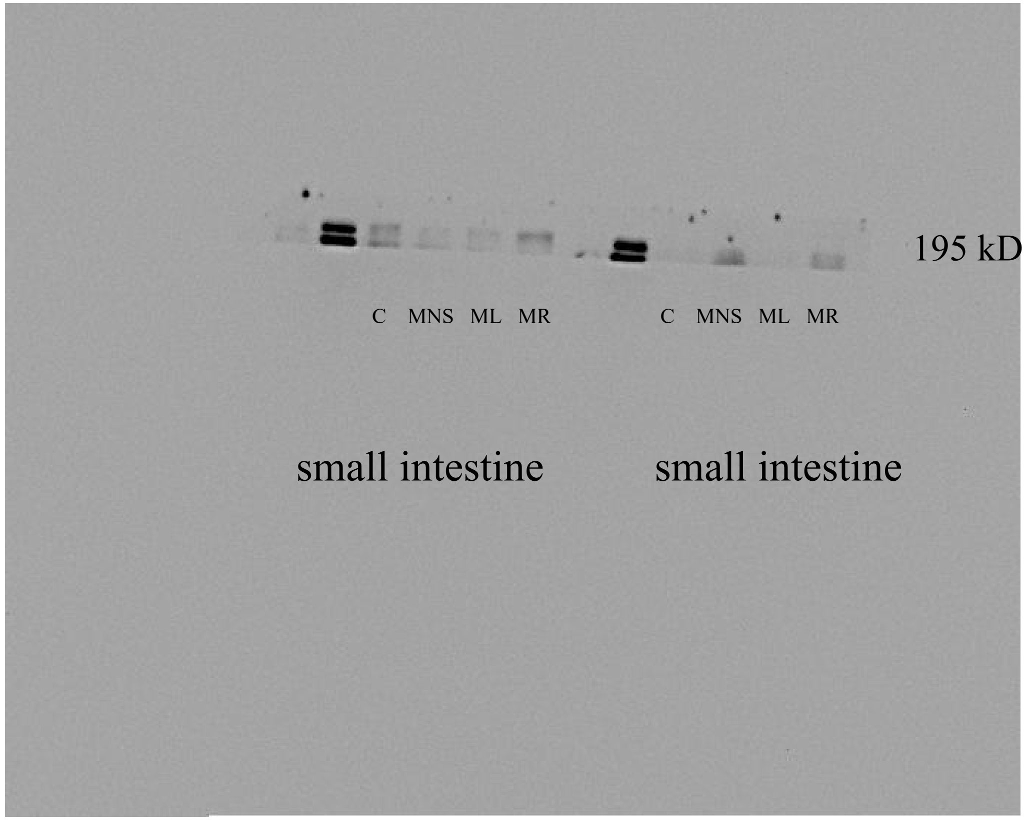

195 kD
C MNS ML MR
C MNS ML MR
small intestine
small intestine

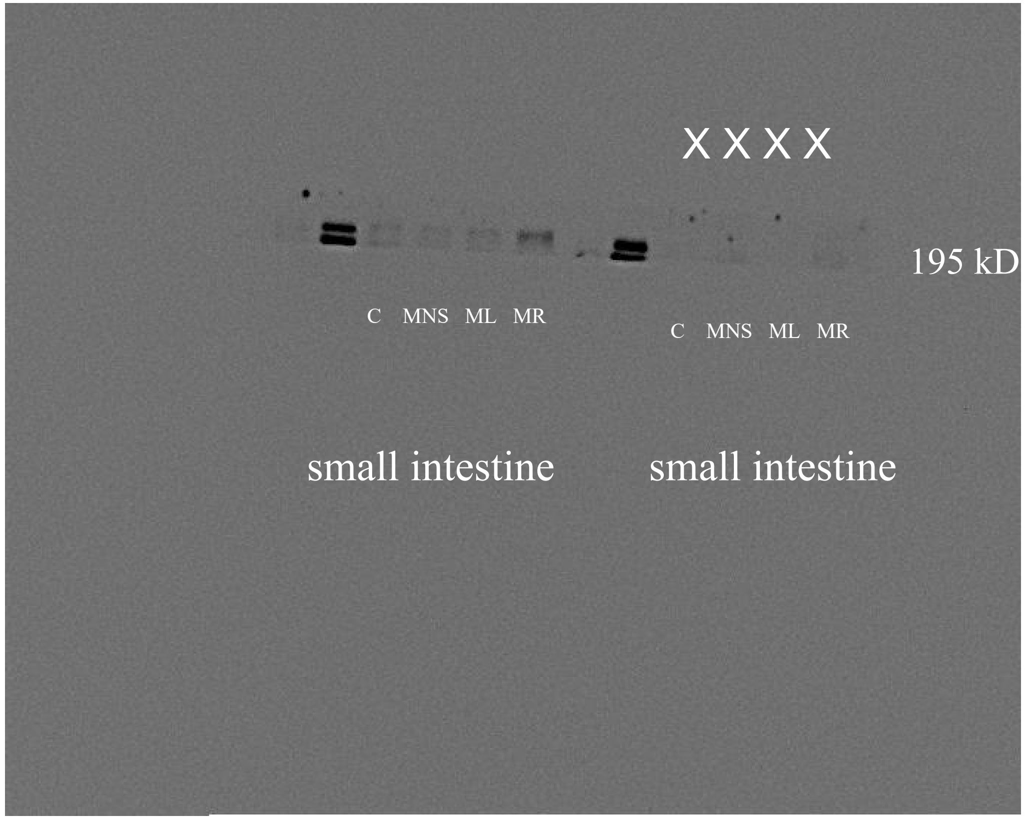

X X X X
195 kD
C MNS ML MR
C MNS ML MR
small intestine
small intestine

Actin

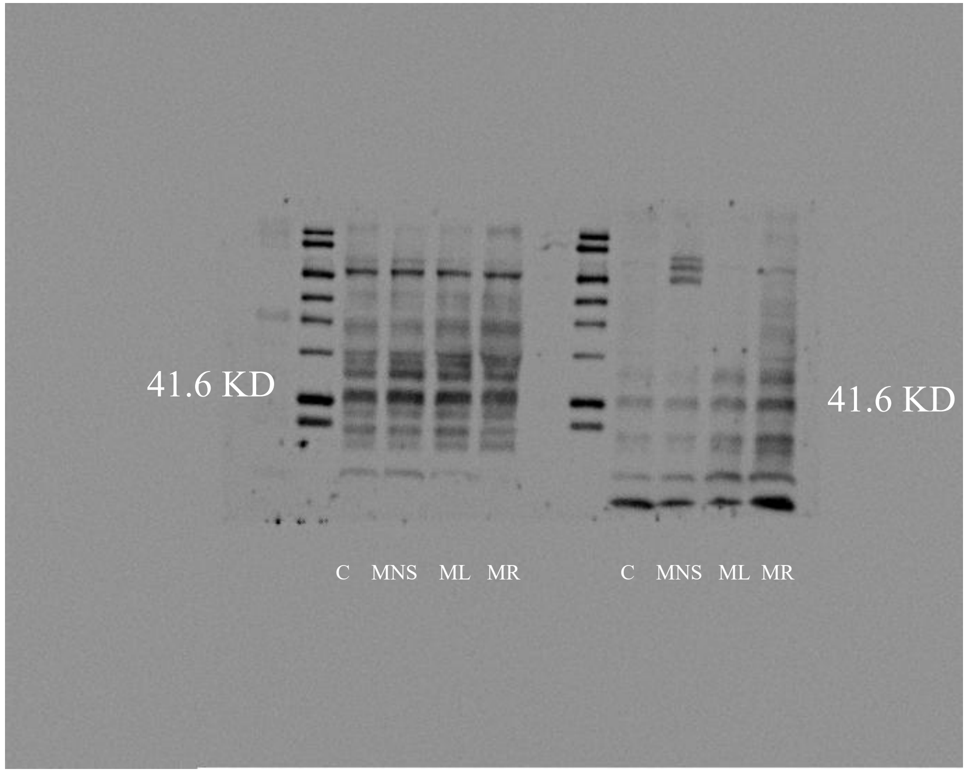
41.6 KD
41.6 KD
C MNS ML MR
C MNS ML MR

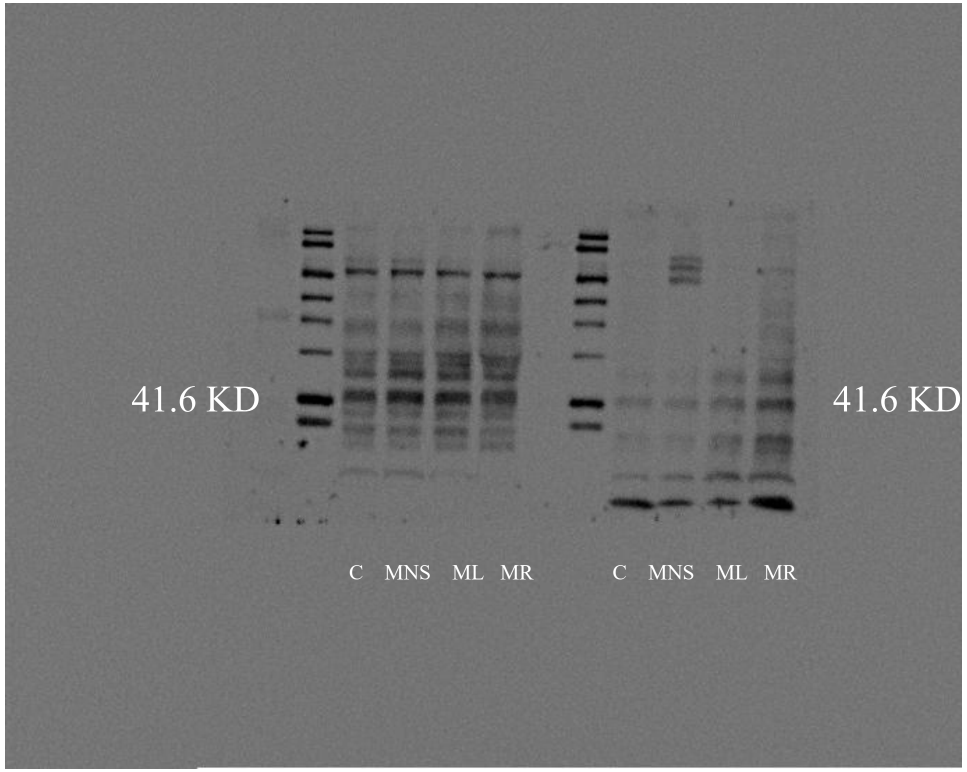
41.6 KD
41.6 KD
C MNS ML MR
C MNS ML MR
