# Supplementary material for: The effects of rifaximin and lactulose on the gut-liver-brain axis in rats with minimal hepatic encephalopathy
Source: PLoS One. 2025 Jun 17;20(6):e0325988. doi: 10.1371/journal.pone.0325988 (PMC12173377; doi:10.1371/journal.pone.0325988)
Supplement: S2 File — (DOCX) [file pone.0325988.s008.docx]

S2 Table. Latency of BAEP I and Escape latencies in four groups after treatment.

| Group | Latency of BAEP I (ms) | Escape latency (s)  (After building, test 4) |
| --- | --- | --- |
| C | 1.28±0.03 | 7.00±4.60 |
| MNS | 1.54±0.05 | 28.43±10.69 |
| ML | 1.35±0.04 | 14.28±6.43 |
| MR | 1.31±0.03 | 8.33±4.82 |

Abbreviations: BAEP, brainstem auditory evoked potentials; MHE, mild hepatic encephalopathy.

S3 Table. Laboratory parameters in four groups after treatment.

| Laboratory parameters | C | MNS | ML | MR |
| --- | --- | --- | --- | --- |
| Serum ammonia (mmol/L) | 0.20±0.02 | 0.45±0.03 | 0.31±0.02 | 0.22±0.02 |
| Cerebrospinal ammonia (mmol/L) | 0.45±0.04 | 0.69±0.15 | 0.52±0.04 | 0.47±0.02 |
| Serum IL-1β (pg/ml) | 3.47±0.10 | 5.57±0.05 | 4.88±0.05 | 3.87±0.13 |
| Serum TNF-α (pg/ml) | 38.21±2.11 | 47.49±0.84 | 43.93±1.43 | 37.49±2.50 |
| Portal LPS (EU/ml) | 0.19±0.05 | 1.67±0.23 | 1.16±0.12 | 1.06±0.15 |
| Liver TLR4 (ng/ml) | 5.95±0.23 | 6.94±0.30 | 6.69±0.21 | 5.97±0.32 |
| Serum ALT (U/L) | 54.30±11.36 | 82.30±7.38 | 77.27±9.20 | 75.57±9.66 |
| Serum AST (U/L) | 224.03±45.23 | 297.88±33.17 | 244.65±25.27 | 242.15±34.45 |
| Serum ALB (g  /L) | 36.65±0.90 | 29.23±0.69 | 30.17±0.69 | 32.83±1.27 |
| Serum TIBL (mmol/L) | 1.23±0.23 | 1.70±0.24 | 1.32±0.26 | 1.42±0.43 |

Abbreviations:IL-1β, interleukin-1β; TNF-α, tumor necrosis factor-α; LPS, lipopolysaccharide; TLR4, toll like receptor 4; ALT, alanine aminotransferase; AST, aspartate aminotransferase; ALB, albumin; TBIL, total bilirubin.

S4 Table. Alpha diversity indices in four groups after treatment.

| Group | Shannon | Simpson |
| --- | --- | --- |
| C | 4.13±0.29 | 0.07±0.04 |
| MNS | 4.44±0.01 | 0.03±0.00 |
| ML | 4.28±0.40 | 0.04±0.01 |
| MR | 3.97±1.00 | 0.09±0.12 |

S5 Table. Differences in microbial functions among groups in pathways of KEGG.

| Group | K02025 | K02026 | K03406 |
| --- | --- | --- | --- |
| C | 0.89±0.36 | 0.85±0.33 | 0.28±0.06 |
| MNS | 0.40±0.08 | 0.40±0.08 | 0.35±0.07 |
| ML | 0.57±0.11 | 0.55±0.10 | 0.18±0.11 |
| MR | 0.42±0.11 | 0.42±0.10 | 0.18±0.04 |

S6 Table. Relative abundance at the genus level in four groups after treatment.

| Genus | C | MNS | ML | MR |
| --- | --- | --- | --- | --- |
| *Akkermansia* | 0.0020±0.0017 | 0.0072±0.0110 | 2.0078±3.4592 | 0.0014±0.0024 |
| *Bacteroides* | 0.3352±0.0137 | 0.2929±0.3617 | 2.0836±0.9594 | 0.1151±0.6696 |
| *Bifidobacterium* | 0.0074±0.0065 | 0.0268±0.0344 | 0.4579±0.4929 | 3.1511±2.1448 |
| *Clostridium_XlVa* | 2.5047±1.1559 | 0.51702±0.3543 | 2.6638±1.6419 | 1.7745±0.9115 |
| *Faecalibacterium* | 0.0000±0.0000 | 0.0011±0.0019 | 0.1804±0.0293 | 0.0000±0.00000 |
| *Lactobacillus* | 26.3329±12.0951 | 4.3735±3.1584 | 2.9449±2.7139 | 31.0146±27.6668 |
| *Prevotella* | 0.1345±0.0573 | 1.5463±0.7677 | 7.3910±5.8611 | 3.849±3.0914 |
| *Ruminococcus* | 1.766±0.6562 | 1.2676±0.6032 | 3.5717±0.5700 | 3.3424±2.2929 |
| *Saccharibacteria* | 2.3445±1.4864 | 14.1543±9.7804 | 0.8356±0.6001 | 2.1287±0.8743 |
| *Streptococcus* | 0.0883±0.0288 | 0.1212±0.1000 | 0.1427±0.0735 | 0.0553±0.0513 |
| *Veillonella* | 0.0009±0.0016 | 0.0518±0.0201 | 0.0742±0.0618 | 0.0071±0.0051 |

S7 Table. Quantitative analysis of Occludin/Actin ggrayscale ratios by Western Blot in rat intestinal tissue across four groups.

| Group | Occludin/Actin 1 | Occludin/Actin 2 | Occludin/Actin 3 |
| --- | --- | --- | --- |
| C | 1.091291 | 1.159969 | 0.851344 |
| MNS | 0.949568 | 0.979755 | 0.787755 |
| ML | 0.973676 | 1.196856 | 0.815222 |
| MR | 1.427612 | 1.620157 | 1.337679 |

S8 Table. Immunohistochemical detection of GABARAP protein expression levels in four groups.

| Group | Relative expression of brain GABARAP |
| --- | --- |
| C | 1.78±0.19 |
| MNS | 2.48±0.12 |
| ML | 1.97±0.19 |
| MR | 2.02±0.15 |

Abbreviations: GABARAP, Gamma-aminobutyric acid receptor-associated protein.
