# Supplementary material for: The effects of rifaximin and lactulose on the gut-liver-brain axis in rats with minimal hepatic encephalopathy
Source: PLoS One. 2025 Jun 17;20(6):e0325988. doi: 10.1371/journal.pone.0325988 (PMC12173377; doi:10.1371/journal.pone.0325988)
Supplement: S3 File — (DOCX) [file pone.0325988.s009.docx]

**Supplementary experimental procedures**

**WB detection**

Take a certain amount of rat colon tissue, add RIPA lysis solution, grind with a tissue grinder, and extract total tissue protein. Centrifuge at 4 ℃ for 10 minutes using a 12000 r/min high-speed centrifuge. Take the supernatant and use the BCA Protein Assay Kit (E-BC-K318-M, Elabscience) to quantify the total protein. After denaturing the protein sample, conduct sodium dodecyl sulfate gel electrophoresis (SDS-PAGE) for 1.5h, and then use 300mA constant flow membrane for 1.0-2.0h. After sealing the PVDF membrane with skim milk powder, the Rabbit Anti ZO-1 (AF5145, Affinity, 1:500), Rabbit Anti Claudin-1 (DF6919, Affinity, 1:500), or Mouse Monoclonal Anti-β ACTIN (TA-09, ZSGB, 1:5000) was incubated overnight at 4 ℃. The next day, the membrane was treated with a secondary antibody on a shaker at room temperature for 1 hour. After the incubation, the membrane was immersed in a chemiluminescent solution and developed using a high-sensitivity chemiluminescence imaging system.

**Immunohistochemistry**

Immunohistochemical staining was performed to detect the expression of γ-aminobutyric acid type A receptor-associated protein (GABARAP) (DF7419, Affinity, 1:100) in the hippocampal region of rat brain tissue. Paraffin-embedded hippocampal sections were baked, deparaffinized, and rehydrated, followed by antigen retrieval using citrate buffer. After blocking with 5% bovine serum albumin (BSA) (A8020, Solarbio), the sections were incubated overnight at 4°C with rabbit anti-GABARAP primary antibody (1:100). The next day, sections were incubated with horseradish peroxidase (HRP)-conjugated goat anti-rabbit secondary antibody, visualized with 3,3'-diaminobenzidine (ZB-2301, ZSGB-BIO, 1:200), and counterstained with hematoxylin. The sections were then dehydrated, cleared, mounted, and examined under a light microscope.
